# Type VIIb secretion systems in Lactobacillales: insights from streptococci and enterococci

**DOI:** 10.1128/jb.00236-26

**Published:** 2026-07-20

**Authors:** Caitlin S. Wiafe-Kwakye, Abigail E. Glenn, Brady L. Spencer

**Affiliations:** 1 Department of Microbiology, Immunology, and Cancer Biology, University of Virginia 174484, Charlottesville, Virginia, USA; University of Notre Dame, Notre Dame, Indiana, USA

**Keywords:** type VII secretion system, interbacterial competition, LXG toxins, *Streptococcus*, *Enterococcus*

## Abstract

The type VII secretion system (T7SS) is an ATPase-powered molecular machine encoded by Actinomycetota (T7SSa) and Bacillota (T7SSb) that exports small alpha-helical effectors with functions in interbacterial competition, host colonization, and virulence. While T7SSb has been well characterized in *Staphylococcus aureus* and *Bacillus subtilis*, its presence and function within the order Lactobacillales have only been thoroughly investigated within the past decade. Experimental evidence for T7SSb function has now been established in select *Streptococcus* and *Enterococcus* species. Here, we review the current understanding of T7SSb in these genera, covering core machinery and secreted substrates, including LXG toxins and WXG100 proteins. We discuss the roles of T7SSb during interbacterial antagonism and host-pathogen interactions, including contributions to cytotoxicity and virulence, as well as mucosal colonization and persistence. We further review known regulators of the T7SSb and evaluate the ecological significance of T7SSb-mediated competition for microbial community stability. Finally, we highlight outstanding questions in the field, including mechanisms of effector delivery, structural determination of the apparatus, impact of effectors on host immunity, and potential therapeutic targeting of T7SSb components.

## INTRODUCTION TO T7SSB IN BACILLOTA

Bacterial secretion systems enable microbes to adapt to their environments through nutrient acquisition, interbacterial competition, and host modulation (1). Broadly conserved mechanisms, including the Sec pathway and the Twin Arginine Translocation (Tat) system, transport unfolded and folded proteins, respectively, across the cytoplasmic membrane in both Gram-positive and Gram-negative bacteria (2, 3). Beyond these general pathways, bacteria encode specialized type-specific secretion systems (T1SS–T11SS) that operate in either one-step or two-step mechanisms to export proteins extracellularly or to inject effectors directly into neighboring bacterial or host cells (1, 4–6). While most of these specialized systems are found in Gram-negative organisms, the Type VII Secretion System (T7SS) is primarily associated with Actinomycetota and Bacillota (7, 8). First identified in *Mycobacterium tuberculosis* through studies of the Region of Difference 1 (RD1) locus, the ESX-1 T7SS was found to utilize the FtsK/SpoIIIE-family ATPase EccC to export small alpha-helical secreted substrates such as ESAT-6 and CFP-10, which are critical to virulence and intracellular survival (9, 10). Since the initial characterization of the ESX-1 system in mycobacteria, related Esx-family systems have been identified in Bacillota, leading to the sub-classification of T7SSa (Actinomycetota, encoding EccC ATPases) and T7SSb (Bacillota, encoding EssC ATPases) (8, 10, 11). Additional unique T7SS ATPases have recently been identified across these phyla and are hypothesized to constitute new T7SS classes, T7SSc–T7SSj (12).

In Gram-positive bacteria, which lack an outer membrane, specialized secretion systems transport effector proteins across the cell envelope, facilitating adaptation to specific niches and competition in polymicrobial environments (1, 13, 14). While the Bacillota T7SSb components lack significant sequence homology with those of the T7SSa, they have some structural and functional similarities. For example, both systems utilize FtsK/SpoIIIE domain-containing ATPases to export alpha-helical WXG100 family proteins (15). Further, T7SSb LXG-motif-containing toxins and their chaperones are structurally reminiscent of the T7SSa PE/PPE proteins (16, 17). In Bacillota, T7b-secreted WXG100 proteins were first associated with virulence in *Staphylococcus aureus* (18–20), and more recently, a role for the T7b-secreted polymorphic LXG toxins in interbacterial competition was demonstrated in *S. aureus* and *Streptococcus intermedius* (17, 21–23).

Although the role of the T7SSb has been well characterized in *S. aureus* over the last two decades, until recently, little was known about the presence and function of T7SSb in Lactobacillales. This order comprises multiple families, including *Aerococcaceae*, *Carnobacteriaceae*, *Enterococcaceae*, *Lactobacillaceae*, and *Streptococcaceae*, and T7SSb appears to be encoded primarily within *Enterococcaceae* and *Streptococcaceae* (unpublished data). *Enterococcus* and *Streptococcus* are often pathobionts that commonly colonize mucosal surfaces and can cause disease particularly in immunocompromised populations (24, 25).

Recent work on T7SSb has revealed functions for this system in interbacterial competition (23, 26, 27), as well as virulence (28–30). These functions likely promote pathobiont persistence in mucosal environments, such as the gastrointestinal and vaginal tracts, as well as the oral cavity. Understanding how T7SS functions in these contexts will reveal new insights into host-pathogen-microbiota interactions and open potential avenues for therapeutic manipulation of these interactions. Here, we review the current knowledge of the T7SSb in *Streptococcus* and *Enterococcus* genera and explore its potential roles in shaping microbial communities and host interactions.

## GENETIC ARCHITECTURE OF T7SSB IN STREPTOCOCCI AND ENTEROCOCCI

### Core machinery

The T7SSb apparatus in Bacillota has been characterized in *S. aureus* and *Bacillus subtilis* and consists of a core set of five machinery components, including the membrane-bound ATPase EssC (YukB), large integral membrane protein EsaA (YueB), membrane-associated pseudokinase EssB (YukC), small membrane protein EssA (YueC), and cytoplasmic adaptor EsaB (YukD) (20, 31, 32). Homologs of these components are encoded in the T7SSb loci of *Streptococcus intermedius*, *Streptococcus agalactiae* (or group B *Streptococcus*, GBS), *Streptococcus gallolyticus*, *Streptococcus suis*, and *Enterococcus faecalis*, suggesting that they constitute the T7SSb apparatus in Lactobacillales as well (7).

EssC is the ATPase driver of numerous T7 secretion systems, and these exhibit diversity in domain architecture (12). The EssC utilized by T7SSb is a large polytopic membrane protein comprising two N-terminal forkhead-associated (FHA) domains responsible for protein–protein interactions with other T7SS machinery components, followed by two transmembrane helices, a domain of unknown function (proposed as D0), and three C-terminal ATPase domains designated D1 through D3 (33, 34). EssC D1 ATPase activity is required for effector secretion in both *S. aureus* and *B. subtilis*, whereas impairment of D2 ATPase activity through mutation of Walker A/B motifs impacted effector secretion in *S. aureus* but not *B. subtilis*, possibly due in part to the role of D2 ATPase activity in *S. aureus* T7SSb membrane complex formation (34, 35). The role of D3 is less clear. Truncated EssC lacking the D3 domain did not support *S. aureus* effector secretion, and D3 contains degenerate Walker B motifs (33, 34, 36). Further, in a solved structure of *Geobacillus thermodenitrificans* D2 and D3, ATP was identified in the binding site of D2, but not D3 (33). This is possibly due to structural occlusion as another study demonstrated that purified *S. aureus* D3 exhibited low binding affinity to ATP, and D3 point mutations could either increase or reduce secretion (36). The role of EssC D3 may not solely depend on ATPase function, as this region is hypervariable and has been proposed to confer substrate specificity, likely via interaction with cognate secreted effectors (37). Because of this, C-terminal D3 divergence in EssC serves as a basis for distinguishing T7SSb subtypes within species. Both *S. aureus* and GBS encode four EssC variants (38, 39), and *L. monocytogenes* harbors up to seven EssC variants (40), with each variant associated with a distinct effector repertoire. This pattern is emerging in other Lactobacillales species as well, with *S. gallolyticus* recently shown to exhibit similar T7SSb heterogeneity (41).

### WXG100 protein EsxA

Canonical substrate EsxA is the most conserved secreted component across T7SS-encoding organisms (10). It belongs to the WXG100 family of small (~100 amino acids) alpha-helical proteins that are capable of homo- and heterodimerization and are defined by a central Trp-x-Gly (WxG) motif (42–45). While these proteins do not have an N-terminal signal sequence, the WxG motifs of the *B. subtilis* YukE (EsxA homolog) and the mycobacterial WXG100 protein EsxE were shown to be important for secretion (45, 46), although, interestingly, mutation of the mycobacterial ESAT-6/EsxA WxG motif (W43R) did not impact its secretion (47). The C-terminal residues of WXG100 substrates often contain YxxxD/E (48) (or weaker HxxxD/ExxHxxxH motifs (42]) that have also been shown to be important for effector secretion in both T7SSa and T7SSb (44–46, 49). Together, the WxG motif and C-terminal residues are proposed to constitute a bipartite recognition signal which targets substrates for export (45). While EsxA across Lactobacillales appears to be structurally conserved, some sequence divergence exists across genera. Percent identity analysis revealed that GBS EsxA1 (strain CJB111) is 65–66% identical to EsxA from *S. suis* (strain GZ0565) and *S. gallolyticus* (strain TX20005), 49% identical to *S. aureus* EsxA (strain USA300_FPR3757), and only 33% identical to EsxA from *E. faecalis* (strain OG1RF) and *S. intermedius* (strain B196) (30). The GBS T7SSb encodes multiple EsxA paralogs. For example, some strains encode two adjacent *esxA* homologs upstream of the T7SSb machinery genes (*esxA1* and *esxA2*, 95% protein identity) and up to two *esxA* genes elsewhere in the genome (approximately 85% identical to those encoded in the T7SSb locus), but it is unclear whether these are functionally redundant (39).

### Variable downstream regions and effector modules

Downstream of the conserved core machinery, T7SSb loci in Lactobacillales encode effector modules consisting of chaperone-, LXG toxin-, DUF4176-, and immunity factor-encoding genes. In *S. aureus*, the WXG100 protein EsxB and WXG100-like proteins EsxC and EsxD serve as chaperones for export of the nuclease EsaD (17). In Lactobacillales, WXG100-like LXG-associated alpha-helical protein (Lap) and DUF4176 chaperones are encoded adjacent to LXG toxin genes and complex with the N-terminal alpha-helical LXG domain to facilitate T7SSb toxin export (16, 50, 51). A conserved FxxxD motif within Lap1 functions as another targeting signal to EssC, analogous to the YxxxD/E C-terminal motif identified in mycobacterial T7SSa substrates (48–50). These LXG proteins contain diverse C-terminal toxin domains and therefore possess a variety of functions, including nuclease, NADase, lipase, ADP-ribosyl cyclase, or membrane-depolarizing activity (21, 23, 52–55). A list of characterized T7SSb LXG toxins in Lactobacillales species is shown in Table 1. Many more Lactobacillales LXG toxins likely exist, and LXG toxin diversity has recently been reported in *S. suis* and *S. gallolyticus* (29, 41). Interestingly, a new class of LXG toxins was identified in *S. aureus* that have a reverse arrangement, with the LXG domain located at the C-terminus and the phospholipase toxin domain at the N-terminus; homologs of these reverse LXG toxins were also observed in *E. faecalis* (54).

**TABLE 1:** Characterized Lactobacillales LXG toxins

LXG toxin	Species	Function	Immunity factor	Reference
TelA	*S. intermedius*	Unknown	TipA	(23)
TelB		NADase	TipB	
TelC		Lipid II phosphatase	TipC	
TelD		Unknown	TipD	(50)
OG1RF_11121	*E. faecalis*	Unknown	OG1RF_11122	(26)
TelE	*S. gallolyticus*	Membrane depolarization	TipE	(56)
BltA	*S. agalactiae*	Unknown	Unknown	(27)
LXG1-CT3	*S. suis*	Membrane depolarization	LXG-CT3/Imm3	(29)
Tac1	*S. parasanguinis*	ADP-ribosyl cyclase	Tci1	(55)

“Complete” LXG modules typically consist of genes encoding Lap chaperones, an LXG toxin, an immunity factor, and often a DUF4176 domain-containing protein. Some T7SSb loci contain additional incomplete LXG modules farther downstream that encode fragments of LXG toxins (26, 39). In *S. suis*, these fragments (termed Minimal Substrate Export Toxins (MSE-ExTs)) were recently identified downstream of truncated EssC variants (57). Variable regions of T7SSb loci have been shown to be sites of recombination due to the presence of paralogous genes (58). It is possible that during recombination events these fragments may be reconstituted with an N-terminal LXG domain resulting in a functional toxin (53), potentially analogous to what was observed for Rhs toxins (59); however, this has not yet been experimentally demonstrated. LXG modules and WXG100 genes are also commonly encoded outside of the primary T7SS locus (23, 29, 39, 41, 60).

## FUNCTIONS OF T7SSB IN LACTOBACILLALES

### Interbacterial competition

The T7SSb exports LXG toxins that are increasingly recognized to promote interbacterial competition in both mucosal and environmental niches (23, 61, 62). In *B. subtilis*, T7SSb toxins and immunity factor pairs were hypothesized to influence community composition in soil-associated environments and in biofilms (63). Further, metagenomic analysis of human gut microbiome data demonstrated that LXG toxins are encoded broadly by host- and agriculture-associated bacterial species in Clostridiales, Bacillales, and Lactobacillales (23). Therefore, T7SSb and their exported LXG toxins likely shape polymicrobial communities in both the host and environment.

In the past decade, several LXG toxins have been identified in Lactobacillales, and some of these have been experimentally demonstrated to antagonize prey bacteria *in vitro* (22, 26, 27, 64). The most mechanistic work on LXG toxin secretion and function has been performed in *Streptococcus intermedius*, which encodes TelA, TelB, TelC, and TelD. Each toxin is encoded adjacent to cognate Lap chaperones, which are required for export, and cognate immunity factors, which prevent self-intoxication (16, 50). Many LXG toxins are additionally encoded near large DUF4176 chaperones (or “LXG toxin chaperones,” Ltc), which facilitate toxin stability and export by interacting with the toxin’s middle intrinsically disordered domain (e.g., TelA and TelB). TelC, which lacks this middle domain, has no associated Ltc (51). Of these *S. intermedius* toxins, the functions of TelA and TelD remain undefined, TelB is an NADase, and TelC disrupts cell wall synthesis by degrading lipid II (23, 50).

Outside of *S. intermedius*, several additional Lactobacillales LXG toxins have been described in the last few years. First, the *Streptococcus parasanguinis* LXG toxin Tac1 contains a C-terminal ADP-ribosyl cyclase/hydrolase (ARC) domain and was shown to deplete NAD^+^ and NADP^+^ to restrict bacterial growth (55). In *S. gallolyticus*, the LXG toxin TelE contains a glycine zipper motif within its C-terminal domain and exerts membrane-depolarizing activity, which is mitigated by an adjacently encoded transmembrane domain-containing immunity factor (56). LXG-mediated competition has also recently been studied in *Streptococcus suis*. Similar to that observed in *S. intermedius*, *S. suis* LXG toxin LXG1-CT3 secretion was dependent on a DUF4176 domain-containing protein (29). Further, LXG1-CT3 toxicity was mediated by bacterial membrane damage, as observed for *S. gallolyticus* TelE. Secretion of these LXG toxins is hypothesized to facilitate niche competition in pig tonsils, where multiple *S. suis* strains may co-colonize (64). In GBS, T7SSb was shown to facilitate interbacterial antagonism through LXG toxin BltA, reducing recovery of pathobiont *Enterococcus faecalis* during *in vitro* co-culture and during *in vivo* murine vaginal co-colonization (27). Induced expression of BltA in *E. coli* also resulted in significant toxicity that was dependent on co-expression of an alpha-helical chaperone, either BlpA1 or BlpA2, although the function of BltA remains unknown. Finally, the *E. faecalis* T7SS has also been shown to promote interbacterial competition with non-streptococcal Gram-positive bacteria upon induction of T7SSb following phage infection. This was shown to be mediated by an LXG toxin encoded by OG1RF_11121, and this competition was abrogated by expression of its cognate immunity factor OG1RF_11122 in prey bacteria (26).

### Impact on virulence and host responses

Beyond interbacterial antagonism, T7SSb in Lactobacillales also contributes to host interactions and virulence. WXG100 proteins, such as the ubiquitous T7SS effector EsxA, have been shown in multiple species to promote pathogenesis. In the first studies published on T7SSb in Bacillota, Burts et al. demonstrated that WXG100 protein secretion promotes *S. aureus* abscess formation (18, 19). EsxA was also shown to promote virulence in *S. suis* models of infection (65), and in *Streptococcus gordonii*, EsxA was recently shown to induce neutrophil extracellular trap formation, which contributes to vegetation formation during infective endocarditis (66). Finally, GBS EsxA was shown to exhibit pore-forming activity in planar lipid bilayers and to promote cytotoxicity in human brain endothelial cells in a WxG-motif-dependent manner (30).

Some T7SSb LXG toxins may contribute to virulence and/or modulation of host responses during infection. In *S. aureus*, for example, EsxX was shown to promote virulence and immune evasion due to lysis of neutrophils (28). While the impact of many known Lactobacillales LXG toxins on virulence and/or host responses has not been studied, the *S. suis* LXG1-CT3 and LXG-2 were both recently shown to promote pathogenesis during an intraperitoneal murine infection (29, 67). Our current unpublished work suggests that LXG toxin BltA in GBS does not contribute to pathogenesis during an adult model of hematogenous meningitis, in both clinical scoring of disease severity and tissue bacterial burdens, possibly because this model bypasses initial GBS seeding of a mucosal surface.

### Impact of T7SSb on bacterial physiology

Finally, it is possible that T7SSb in Lactobacillales serves additional physiological roles beyond interbacterial antagonism and virulence. For example, the T7SS was shown to function in sporulation in *Bacillus cereus* and development of *Streptomyces scabies* (68, 69), as well as iron acquisition in *Mycobacterium tuberculosis* (70). Interestingly, in plant commensal *Bacillus velezensis*, T7SSb was shown to co-opt iron from plant roots following YukE/EsxA-mediated plant membrane permeabilization, thereby promoting bacterial root colonization (62). Similar functions have yet to be confirmed for T7SSb in Lactobacillales but may vary depending on microbe-specific ecological conditions.

## REGULATION AND INDUCING CONDITIONS

The regulation of T7SSb varies across Gram-positive bacteria. In *S. aureus*, the alternative sigma factor SigB serves as a complex regulator of *esxA*, activating ArlRS and SpoVG to drive *esxA* transcription while also promoting expression of SarA, which represses *esxA* (71, 72). In addition to these regulators, host-derived *cis*-unsaturated fatty acids (including linoleic acid) have been shown to activate the *S. aureus* (USA300) T7SS by incorporating into the bacterial membrane via the fatty acid kinase complex, thereby decreasing bacterial membrane fluidity (73). Growth of *S. aureus* (RN6390) in media containing hemoglobin, hemin, or CaCl_2_ has also been shown to stimulate T7 secretion (74). In *Bacillus subtilis*, T7SS expression appears to be coordinated with developmental processes, particularly sporulation-linked regulatory programs (75). The pleiotropic regulator DegU activates the *yfj* (T7SSb) operon in cells entering sporulation, whereas in later stages of sporulation, rising Spo0A levels repress the operon (75).

In *E. faecalis*, regulation of T7SS was shown to depend on the membrane-damage-sensing protein IreK, as well as the putative GntR transcriptional regulator OG1RF_11099, which is encoded directly upstream of the T7SS locus (26). This enterococcal T7SS was further shown to be induced under conditions of cellular stress, such as phage infection or exposure to sub-lethal concentrations of cell envelope-targeting antibiotics, resulting in toxin-mediated competition against non-kin bacteria (26). Our current understanding of T7SSb regulation in streptococci is much more limited. In GBS, a direct regulator of T7SS expression has not been identified, but T7SS genes were observed to be dysregulated in a mutant lacking the CRISPR-associated endonuclease Cas9 (76). Previous RT-PCR analysis suggests that GBS T7SS-I *esxA1* is independently transcribed, while the remainder of the T7SS-I locus may be co-transcribed (39), reminiscent of the dedicated *esxA* promoter identified in *S. aureus* (20). It is unclear if GBS *esxA1* is regulated independently of *esxA2* and the remainder of the GBS T7SS-I locus. Furthermore, a putative promoter has been identified within the 3′ region of the GBS subtype I *essC* gene (unpublished work), raising the possibility that parts of the T7SS locus may be differentially expressed depending on environmental conditions or host niches, although this needs to be verified experimentally.

**Fig 1:**
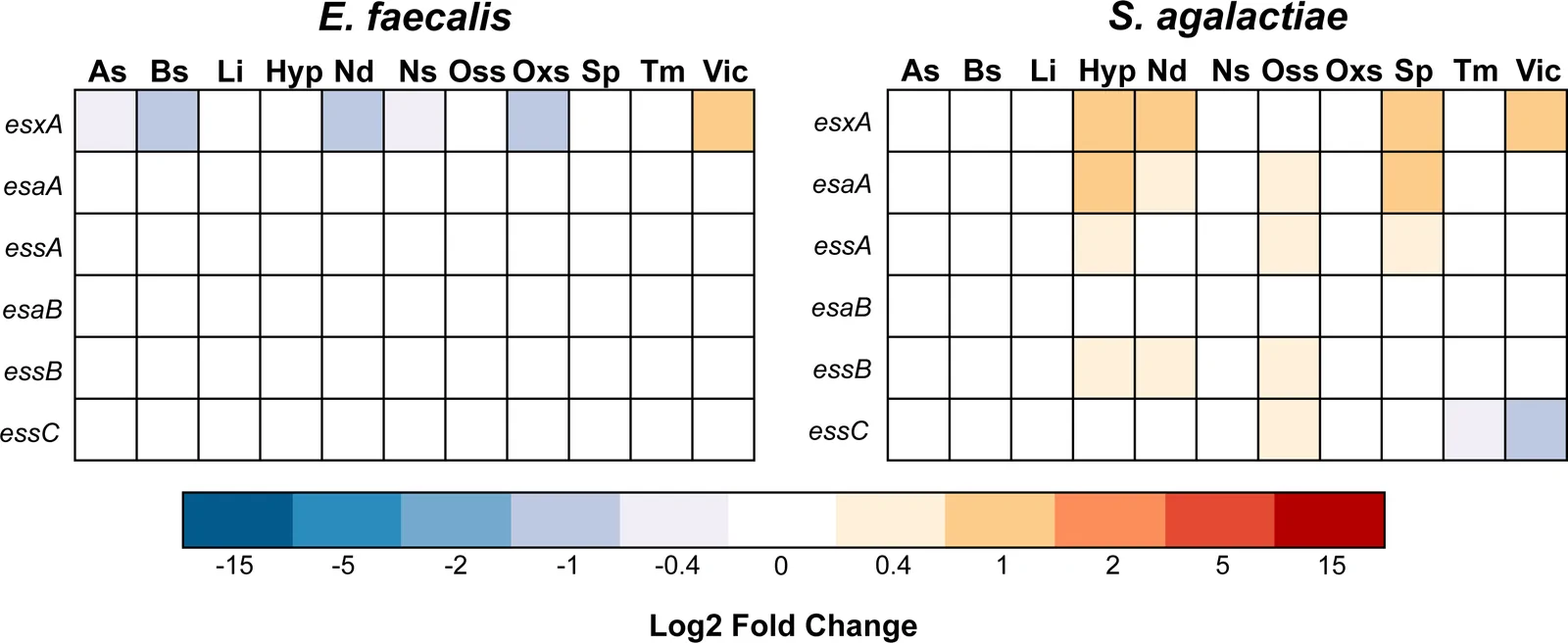
Differential expression of T7SS genes in *S. agalactiae* and *E. faecalis* under various stress conditions. Heatmap showing significant (FDR-corrected *P*-value < 0.05) *esxA* expression when subjected to a variety of environmental stressors. Heatmap generated from PATHOgenex. Stressors tested are acid stress (As), bile stress (Bs), low iron (Li), hypoxia (Hyp), nutritional downshift (Nd), nitrosative stress (Ns), osmotic stress (Oss), oxidative stress (Oxs), stationary phase (Sp), temperature (Tm), and virulence-inducing condition (Vic). Values indicate log_2_ fold change. *E. faecalis* strain OG1RF and *S. agalactiae* strain CCU42 were used. GBS reads were mapped to the NEM316 reference genome.

To assess environmental conditions that might impact T7SSb expression, we utilized publicly available data sets from Avican et al. via the open-access interactive RNA atlas, PATHOgenex, which defined transcriptomic responses of 32 bacterial pathogens across 11 infection-related stress conditions (77). Five of these 32 pathogens are within the Lactobacillales order; however, neither group A *Streptococcus*, *Streptococcus pneumoniae*, nor the *S. suis* strain used in the study (P1/7) encodes T7SSb (78). As such, we assessed T7SS gene expression in the two remaining species, *S. agalactiae* (strain CCU42) and *E. faecalis* (OG1RF). Interestingly, *E. faecalis esxA* expression was solely upregulated in “virulence-inducing conditions” (Vic; indicated as growth in 10% fetal bovine serum for four hours), and no significant changes were observed in the T7SS machinery genes (*esaA–essC*) in any condition tested (Fig. 1) (77). In contrast, the *S. agalactiae esxA* was upregulated in multiple stress conditions, including hypoxia, nutritional downshift, stationary phase, and virulence-inducing conditions (77). Some *S. agalactiae* T7SS machinery genes were also induced in hypoxia, nutritional downshift, and stationary phase, but not in virulence-inducing conditions (in contrast to *esxA*). As mentioned above, more experimental work is needed to determine if *esxA* is regulated differently from the T7SS machinery genes in Lactobacillales. Finally, upon mining published RNA-seq data sets, T7SSb gene expression in *S. agalactiae* appears to be induced in the presence of mucins (79) and amniotic fluid (80), but not in the murine vaginal lumen (81, 82), human U937 monocytes (83), or during zinc intoxication (84). Therefore, T7SSb induction is likely species-specific and restricted to specific ecological pressures.

## OUTSTANDING QUESTIONS WITHIN THE LACTOBACILLALES T7SSB FIELD

Because most of the literature on T7SSb in Lactobacillales has emerged in the last decade, several fundamental questions remain about its ecological importance, underlying export mechanism, structure, and functions.

### Ecological significance of Lactobacillales T7SSb

As described above, many *Streptococcus* and *Enterococcus* species are pathobionts that colonize the oral, vaginal, and gastrointestinal mucosae, where microbial community stability is critical for health. While T7SSb-mediated interbacterial competition has been demonstrated *in vitro* in several species, its impact on microbial community structure in these mucosal niches *in vivo* remains largely unexplored. A key limitation of existing competition studies is that the predator-prey pairs examined *in vitro* do not always represent physiologically relevant antagonistic interactions between species that co-inhabit the same niche *in vivo*. Moving forward, it will therefore be important to establish T7SSb-mediated competition between ecologically relevant competitors in relevant *in vivo* models.

Mucosal dysbiosis can result from changes in host physiology that alter the metabolic landscape of mucosal surfaces, creating selective niches that favor the expansion of specific taxa (85). In addition to direct competition with microbiota members, the T7SSb may also shape microbial community composition indirectly by altering the host metabolic environment through epithelial barrier damage and inflammation, similar to mechanisms described for other pathogens (86, 87). The impact of T7SSb-mediated interbacterial antagonism on microbial communities is likely species-, subtype-, and niche-dependent. In some contexts, the T7SSb may serve a homeostatic function, promoting colonization resistance in mucosal niches; in others, it may disrupt microbiota stability, resulting in disease. For example, disruption of the vaginal microbiota is associated with bacterial vaginosis and aerobic vaginitis, both of which are established risk factors for preterm birth and adverse reproductive outcomes (88, 89). Mining of clinical microbiome data sets will therefore be important to determine whether the presence of specific T7SSb subtypes or toxins correlates with altered microbial community compositions and/or disease states.

The impact of specific T7SS subtypes on GBS vaginal colonization provides a relevant example of this complexity. Four T7SSb subtypes have been identified in *S. agalactiae*, each encoding a unique effector repertoire, and these subtypes differ in their contribution to vaginal colonization by their respective representative clinical isolates (39). Deletion of *essC* in the hyper-persistent subtype I isolate CJB111 reduced GBS persistence in the murine vaginal tract, whereas *essC* deletion in the subtype III isolate CNCTC 10/84 unexpectedly enhanced GBS persistence (39). We hypothesize that subtype III effectors activate host responses that promote GBS clearance and that the rarity of subtype III among vaginal isolates may reflect this selective pressure (39). These data suggest that different T7SSb effector repertoires, even within the same species, can promote differing ecological outcomes during colonization.

### Effector delivery mechanism and target specificity

While the molecular requirements for export of some effectors have been defined, the mechanism by which T7SSb effectors are delivered into a recipient cell remains unknown. Contact-dependent killing has been demonstrated for T7SSb in a few species (23, 26, 30), but whether this reflects direct injection through an extracellular apparatus, diffusion of secreted effectors following contact, and/or engagement of a surface receptor on the prey cell has not been established. EsxA has been hypothesized to function as a core component of the T7SS machinery, largely based on work in *S. aureus* in which EsxA was co-isolated from membrane fractions along with other T7SSb structural components (90). Additionally, EsxA was shown to be necessary for secretion of other effectors (44). Because of this and because of EsxA’s demonstrated pore-forming activity (30), it is tempting to speculate that EsxA may function as a translocon component. Indeed, WXG100 proteins have been hypothesized to function as channel-forming components in T7SSa (46). If EsxA pore-forming activity evolved to facilitate T7SS effector delivery, then its damage to the host may represent “accidental” collateral damage. This is corroborated by the fact that T7SS, including EsxA homologs, are encoded by both pathogenic and non-pathogenic organisms (11). As such, the loss of virulence observed upon *esxA* deletion described above could reflect a direct role for EsxA in pathogenesis and/or an indirect effect resulting from impaired secretion of other T7SSb effectors that depend on EsxA for export.

It is also unclear what dictates T7SSb susceptibility of prey bacteria. One hypothesis is that susceptibility is determined simply by the absence of the cognate immunity factor. In this model, T7SSb indiscriminately “fires” into all cells, and only those that have the cognate immunity factor survive. However, we previously observed a gradient of *E. faecalis* isolate susceptibility to the GBS T7SS-I (27), suggesting that susceptibility may not be binary. Another hypothesis is that T7SS-mediated competition may be governed by cell surface interactions, which convey whether neighboring bacteria are kin or non-kin. In this scenario, T7SS competition would be selective, being initiated only upon detection of neighboring non-kin. Defining the molecular basis of prey susceptibility and whether T7SS “attacks” are targeted (kin versus non-kin discrimination) or indiscriminate are mechanistic gaps in the field. Finally, while LXG toxins are hypothesized to be translocated into a prey cell (63), direct delivery of T7SS effectors during competition has not been experimentally demonstrated.

### Structural resolution of the T7SSb apparatus

The structure of the T7SS machinery complex, in both T7SSa and T7SSb, has proven difficult to resolve, although recent publications have made important advances. In T7SSa, Collars et al. proposed that this full complex is elusive in imaging due to real-time building and disassembly of the apparatus spanning the mycomembrane, which is partially composed of the secreted effectors prior to their export (91). Recent work by Oka et al. in *Bacillus* solved the structure of a portion of the T7SSb apparatus, which is proposed to be linked by the YukD/EsaB cytoplasmic component (31). However, as this structure lacks the second-largest subunit, YueB (EsaA), as well as YueC (EssA), both of which are very likely components of the machinery, more work is needed to complete the structure of the T7SSb apparatus. It is also an open question as to how secreted effectors bypass the thick peptidoglycan layer of these Gram-positive bacteria. The *S. aureus* EssH peptidoglycan hydrolase was shown to be required for assembly of the EsaA machinery component across the cell wall, enabling substrate secretion (92, 93); however, EssH homologs (defined by >50% identity and 50% query cover) do not appear to be encoded in Lactobacillales. CHAP domain-containing proteins with predicted peptidoglycan hydrolase activity can be found in some streptococcal and enterococcal T7SS loci (39), but a functional link to T7SS activity has not been established. Finally, the T7SSb EsaA machinery protein has been proposed to function as an extracellular secretion conduit to facilitate effector export as it is predicted to span the Gram-positive peptidoglycan layer (94). Future work building on these recent advances is needed to determine how the T7SSb apparatus is assembled and how substrates are translocated across the cell envelope, particularly in understudied Lactobacillales.

### Identifying inducing conditions to define the T7SSb secretome

Proteomic approaches such as mass spectrometry have identified T7-secreted proteins in *S. aureus*, *S. intermedius*, *S. gallolyticus*, and recently in *S. suis* (23, 29, 52, 56). However, these methods may not be effective in species where T7SSb is expressed at low levels or is repressed under laboratory conditions, as low-abundance effectors may be missed. An additional challenge to identifying the T7SSb secretome is that substrates can be encoded outside of the T7SSb locus and/or may include hypothetical proteins that are difficult to identify solely by bioinformatic analysis. As discussed above, the conditions that induce T7SS expression in certain species, including GBS, are poorly defined. Defining these signals using transcriptional and fluorescent reporters will help to identify complete species-specific T7SSb secretomes in different environmental conditions.

### T7SS interface with host immunity

WXG100 protein ESAT-6 (EsxA) was first identified in 1995 as a secreted T-cell antigen of *M. tuberculosis* that elicits strong IFN-γ responses (95). There is some evidence of T7SSb-mediated host responses due to colonization or infection by species within the Lactobacillales order. For example, in a systemic infection model, GBS lacking a functional T7SS-I caused less brain inflammation than the parental isolate, consistent with a role for secreted effectors in immune activation (30). The impact of secreted effectors on host responses is less studied during bacterial colonization of mucosal surfaces. While the T7SSb of *S. gallolyticus*, GBS T7SS-I, and *E. faecalis* have been shown to promote mucosal colonization (27, 96, 97), we do not understand the mechanism underlying this enhanced persistence. Our recent work showed that our T7SS subtype I representative GBS isolate does not elicit a cellular immune response during vaginal colonization (98). In our preliminary unpublished work, we observed an increase in vaginal IL-17 in mice colonized with T7SS-I deficient GBS compared to the parental isolate, raising the possibility that T7SS-I may promote immune evasion at mucosal surfaces during asymptomatic colonization; however, it remains unclear which effectors might drive this.

### Potential therapeutic targeting of the T7SSb

The T7SSb represents an underexplored but potentially promising therapeutic target. Small molecule inhibition of secretion system ATPases is an established anti-virulence strategy, with screens against T3SS and T4SS ATPases yielding micromolar inhibitors of ATPase activity, some of which also block effector secretion or associated toxicity (99–101). Because ATPase domain structures of EssC have been solved and the Gram-positive cell wall is permeable to small molecules, EssC inhibitors could be directly screened for. Another strategy involves targeting EsxA for vaccine development. EsxA is known to be immunogenic, and anti-EsxA antibodies were detected in patients with *S. aureus* infection (102). Further, EsxA and EsxB were included in experimental multivalent *S. aureus* vaccines that elicited broad protection in murine models (103, 104). Notably, mutation of the WXG motif has been shown to abolish WXG100 pore-forming activity and/or cytotoxicity (30, 46), raising the possibility that an EsxA variant with a mutated WXG motif could serve as a vaccine candidate that limits host cell damage if its immunogenicity is preserved. Finally, the narrow-spectrum activity and biochemical diversity of LXG toxins could be exploited to develop targeted antimicrobial agents, similar to strategies proposed for bacteriocins (105).

## CONCLUSION

While the T7SSb field in *Streptococcus* and *Enterococcus* is expanding, significant gaps remain in our understanding of T7SSb effector repertoires, regulatory networks, structural assembly, and host interactions. The studies reviewed here establish roles for T7SSb in interbacterial competition, virulence, and host barrier disruption, processes that impact health and disease. Addressing the outstanding questions outlined above will help to define the mechanisms underlying effector export as well as the biological significance of these systems. Future mechanistic T7SSb studies may also identify therapeutic targets for streptococcal and enterococcal infections, for which new strategies are urgently needed.

## References

[1] Green ER, Mecsas J. 2016. Bacterial secretion systems: an overview. Microbiol Spectr 4. doi:10.1128/microbiolspec.VMBF-0012-2015 PMC4804464 26999395

[2] Schneewind O, Missiakas D. 2014. Sec-secretion and sortase-mediated anchoring of proteins in Gram-positive bacteria. Biochim Biophys Acta 1843:1687–1697. doi:10.1016/j.bbamcr.2013.11.009 24269844 PMC4031296

[3] Palmer T, Berks BC. 2024. The twin-arginine translocation (Tat) system. Curr Biol 34:R267–R268. doi:10.1016/j.cub.2024.02.039 38593766

[4] Jamali H, Akrami F, Layeghkhavidaki H, Bouakkaz S. 2025. Bacterial protein secretion systems: mechanisms, functions, and roles in virulence. Microb Pathog 206:107790. doi:10.1016/j.micpath.2025.107790 40480450

[5] Gupta R, Gupta N. 2021. Protein secretion, p 235–264. *In* Gupta R, Gupta N (ed), Fundamentals of bacterial physiology and metabolism. Springer, Singapore.

[6] Filloux A. 2022. Bacterial protein secretion systems: game of types: this article is part of the bacterial cell envelopes collection. Microbiology 168. doi:10.1099/mic.0.001193 35536734

[7] Spencer BL, Doran KS. 2022. Evolving understanding of the type VII secretion system in Gram-positive bacteria. PLoS Pathog 18:e1010680. doi:10.1371/journal.ppat.1010680 35901012 PMC9333272

[8] Unnikrishnan M, Constantinidou C, Palmer T, Pallen MJ. 2017. The enigmatic Esx proteins: looking beyond mycobacteria. Trends Microbiol 25:192–204. doi:10.1016/j.tim.2016.11.004 27894646

[9] Stanley SA, Raghavan S, Hwang WW, Cox JS. 2003. Acute infection and macrophage subversion by *Mycobacterium tuberculosis* require a specialized secretion system. Proc Natl Acad Sci USA 100:13001–13006. doi:10.1073/pnas.2235593100 14557536 PMC240734

[10] Abdallah AM, Gey van Pittius NC, DiGiuseppe Champion PA, Cox J, Luirink J, Vandenbroucke-Grauls CMJE, Appelmelk BJ, Bitter W. 2007. Type VII secretion — mycobacteria show the way. Nat Rev Microbiol 5:883–891. doi:10.1038/nrmicro1773 17922044

[11] Pallen MJ. 2002. The ESAT-6/WXG100 superfamily -- and a new Gram-positive secretion system? Trends Microbiol 10:209–212. doi:10.1016/s0966-842x(02)02345-4 11973144

[12] Garrett SR, Higginson AB, Palmer T. 2024. Multiple variants of the type VII secretion system in Gram-positive bacteria. microLife 5:uqae013. doi:10.1093/femsml/uqae013 38957458 PMC11217815

[13] Maphosa S, Moleleki LN, Motaung TE. 2023. Bacterial secretion system functions: evidence of interactions and downstream implications. Microbiology (Reading) 169:001326. doi:10.1099/mic.0.001326 37083586 PMC10202321

[14] Tseng T-T, Tyler BM, Setubal JC. 2009. Protein secretion systems in bacterial-host associations, and their description in the gene ontology. BMC Microbiol 9 Suppl 1:S2. doi:10.1186/1471-2180-9-S1-S2 19278550 PMC2654662

[15] Tran HR, Grebenc DW, Klein TA, Whitney JC. 2021. Bacterial type VII secretion: an important player in host‐microbe and microbe‐microbe interactions. Mol Microbiol 115:478–489. doi:10.1111/mmi.14680 33410158

[16] Klein TA, Shah PY, Gkragkopoulou P, Grebenc DW, Kim Y, Whitney JC. 2024. Structure of a tripartite protein complex that targets toxins to the type VII secretion system. Proc Natl Acad Sci USA 121:e2312455121. doi:10.1073/pnas.2312455121 38194450 PMC10801868

[17] Yang Y, Boardman E, Deme J, Alcock F, Lea S, Palmer T. 2023. Three small partner proteins facilitate the type VII-dependent secretion of an antibacterial nuclease. mBio 14:e02100–23. doi:10.1128/mbio.02100-23 37815362 PMC10653861

[18] Burts ML, Williams WA, DeBord K, Missiakas DM. 2005. EsxA and EsxB are secreted by an ESAT-6-like system that is required for the pathogenesis of *Staphylococcus aureus* infections. Proc Natl Acad Sci USA 102:1169–1174. doi:10.1073/pnas.0405620102 15657139 PMC545836

[19] Burts ML, DeDent AC, Missiakas DM. 2008. EsaC substrate for the ESAT-6 secretion pathway and its role in persistent infections of *Staphylococcus aureus*. Mol Microbiol 69:736–746. doi:10.1111/j.1365-2958.2008.06324.x 18554323 PMC2597432

[20] Kneuper H, Cao ZP, Twomey KB, Zoltner M, Jäger F, Cargill JS, Chalmers J, van der Kooi-Pol MM, van Dijl JM, Ryan RP, Hunter WN, Palmer T. 2014. Heterogeneity in ess transcriptional organization and variable contribution of the Ess/Type VII protein secretion system to virulence across closely related *Staphyloccus aureus* strains. Mol Microbiol 93:928–943. doi:10.1111/mmi.12707 25040609 PMC4285178

[21] Cao Z, Casabona MG, Kneuper H, Chalmers JD, Palmer T. 2016. The type VII secretion system of *Staphylococcus aureus* secretes a nuclease toxin that targets competitor bacteria. Nat Microbiol 2:16183. doi:10.1038/nmicrobiol.2016.183 27723728 PMC5325307

[22] Boardman ER, Palmer T, Alcock F. 2023. Interbacterial competition mediated by the type VIIb secretion system. Microbiology (Reading) 169:001420. doi:10.1099/mic.0.001420 38116759 PMC10765036

[23] Whitney JC, Peterson SB, Kim J, Pazos M, Verster AJ, Radey MC, Kulasekara HD, Ching MQ, Bullen NP, Bryant D, Goo YA, Surette MG, Borenstein E, Vollmer W, Mougous JD. 2017. A broadly distributed toxin family mediates contact-dependent antagonism between gram-positive bacteria. eLife 6:e26938. doi:10.7554/eLife.26938 28696203 PMC5555719

[24] Sangiorgio G, Calvo M, Migliorisi G, Campanile F, Stefani S. 2024. The impact of *Enterococcus* spp. in the immunocompromised host: a comprehensive review. Pathogens 13:409. doi:10.3390/pathogens13050409 38787261 PMC11124283

[25] Nobbs AH, Lamont RJ, Jenkinson HF. 2009. *Streptococcus adherence* and colonization. Microbiol Mol Biol Rev 73:407–450, doi:10.1128/MMBR.00014-09 19721085 PMC2738137

[26] Chatterjee A, Willett JLE, Dunny GM, Duerkop BA. 2021. Phage infection and sub-lethal antibiotic exposure mediate *Enterococcus faecalis* type VII secretion system dependent inhibition of bystander bacteria. PLoS Genet 17:e1009204. doi:10.1371/journal.pgen.1009204 33411815 PMC7790226

[27] Job AM, Doran KS, Spencer BL. 2024. A group B streptococcal type VII-secreted LXG toxin mediates interbacterial competition and colonization of the murine female genital tract. mBio 15. doi:10.1128/mbio.02088-24 PMC11481500 39189749

[28] Dai Y, Wang Y, Liu Q, Gao Q, Lu H, Meng H, Qin J, Hu M, Li M. 2017. A novel ESAT-6 secretion system-secreted protein EsxX of community-associated *Staphylococcus aureus* lineage ST398 contributes to immune evasion and virulence. Front Microbiol 8. doi:10.3389/fmicb.2017.00819 PMC5418362 28529509

[29] Wu H, Wu Y, Bai Q, Liang Z, Zhu X, Pan X, Liu M, Yao H, Ma J, Wu Z. 2025. A LXG toxin stabilized by DUF4176 contributes to *Streptococcus suis* competition and pathogenicity. BMC Biol 23:284. doi:10.1186/s12915-025-02391-9 41024071 PMC12482557

[30] Spencer BL, Tak U, Mendonça JC, Nagao PE, Niederweis M, Doran KS. 2021. A type VII secretion system in group B *Streptococcus* mediates cytotoxicity and virulence. PLoS Pathog 17:e1010121. doi:10.1371/journal.ppat.1010121 34871327 PMC8675928

[31] Oka GU, Benoit N, Siroy A, Gubellini F, Marza E, Fronzes R. 2025. A ubiquitin-like protein controls assembly of a bacterial type VIIb secretion system. Sci Adv 11:eady9587. doi:10.1126/sciadv.ady9587 41270170 PMC12637312

[32] Tassinari M, Doan T, Bellinzoni M, Chabalier M, Ben-Assaya M, Martinez M, Gaday Q, Alzari PM, Cascales E, Fronzes R, Gubellini F. 2022. The antibacterial type VII secretion system of *Bacillus subtilis*: structure and interactions of the pseudokinase YukC/EssB. mBio 13. doi:10.1128/mbio.00134-22 PMC9600267 36154281

[33] Zoltner M, Ng WMAV, Money JJ, Fyfe PK, Kneuper H, Palmer T, Hunter WN. 2016. EssC: domain structures inform on the elusive translocation channel in the type VII secretion system. Biochem J 473:1941–1952. doi:10.1042/BCJ20160257 27130157 PMC4925161

[34] Bobrovskyy M, Oh SY, Missiakas D. 2022. Contribution of the EssC ATPase to the assembly of the type 7b secretion system in *Staphylococcus aureus**.* J Biol Chem 298:102318. doi:10.1016/j.jbc.2022.102318 35921891 PMC9436818

[35] Ramsdell TL, Huppert LA, Sysoeva TA, Fortune SM, Burton BM. 2015. Linked domain architectures allow for specialization of function in the FtsK/SpoIIIE ATPases of ESX secretion systems. J Mol Biol 427:1119–1132. doi:10.1016/j.jmb.2014.06.013 24979678 PMC4277743

[36] Mietrach N, Damián-Aparicio D, Mielich-Süss B, Lopez D, Geibel S. 2020. Substrate interaction with the EssC coupling protein of the type VIIb secretion system. J Bacteriol 202:e00646-19. doi:10.1128/JB.00646-19 31964696 PMC7167477

[37] Jäger F, Kneuper H, Palmer T. 2018. EssC is a specificity determinant for *Staphylococcus aureus* type VII secretion. Microbiology 164:816–820. doi:10.1099/mic.0.000650 29620499 PMC5994694

[38] Warne B, Harkins CP, Harris SR, Vatsiou A, Stanley-Wall N, Parkhill J, Peacock SJ, Palmer T, Holden MTG. 2016. The Ess/Type VII secretion system of *Staphylococcus aureus* shows unexpected genetic diversity. BMC Genomics 17:222. doi:10.1186/s12864-016-2426-7 26969225 PMC4788903

[39] Spencer BL, Job AM, Robertson CM, Hameed ZA, Serchejian C, Wiafe-Kwakye CS, Mendonça JC, Apolonio MA, Nagao PE, Neely MN, Korotkova N, Korotkov KV, Patras KA, Doran KS. 2023. Heterogeneity of the group B streptococcal type VII secretion system and influence on colonization of the female genital tract. Mol Microbiol 120:258–275. doi:10.1111/mmi.15115 37357823 PMC10527989

[40] Bowran K, Garrett SR, van Vliet AHM, Palmer T. 2023. A novel variant of the *Listeria monocytogenes* type VII secretion system EssC component is associated with an Rhs toxin. Microb Genom 9. doi:10.1099/mgen.0.001036 PMC10327511 37278699

[41] Calderon G, Tamang J, Woodfin S, Prah I, Hurdle J, Xu Y. 2026. Systematic analysis of the type VII secretion system in *Streptococcus gallolyticus* subsp. *gallolyticus* reveals genomic diversity and functional associations. Front Microbiol 17:1844499. doi:10.3389/fmicb.2026.1844499 42293541 PMC13253547

[42] Poulsen C, Panjikar S, Holton SJ, Wilmanns M, Song Y-H. 2014. WXG100 protein superfamily consists of three subfamilies and exhibits an α-Helical C-terminal conserved residue pattern. PLoS One 9:e89313. doi:10.1371/journal.pone.0089313 24586681 PMC3935865

[43] Shukla A, Pallen M, Anthony M, White SA. 2010. The homodimeric GBS1074 from *Streptococcus agalactiae*. Acta Crystallogr F Struct Biol Cryst Commun 66:1421–1425. doi:10.1107/S1744309110036286 PMC3001639 21045286

[44] Anderson M, Aly KA, Chen YH, Missiakas D. 2013. Secretion of atypical protein substrates by the ESAT-6 secretion system of *Staphylococcus aureus**.* Mol Microbiol 90:734–743. doi:10.1111/mmi.12395 24033479 PMC3951145

[45] Sysoeva TA, Zepeda-Rivera MA, Huppert LA, Burton BM. 2014. Dimer recognition and secretion by the ESX secretion system in *Bacillus subtilis*. Proc Natl Acad Sci USA 111:7653–7658. doi:10.1073/pnas.1322200111 24828531 PMC4040557

[46] Tak U, Dokland T, Niederweis M. 2021. Pore-forming Esx proteins mediate toxin secretion by *Mycobacterium tuberculosis**.* Nat Commun 12:394. doi:10.1038/s41467-020-20533-1 33452244 PMC7810871

[47] Brodin P, de Jonge MI, Majlessi L, Leclerc C, Nilges M, Cole ST, Brosch R. 2005. Functional analysis of early secreted antigenic target-6, the dominant T-cell antigen of *Mycobacterium tuberculosis*, reveals key residues involved in secretion, complex formation, virulence, and immunogenicity. J Biol Chem 280:33953–33959. doi:10.1074/jbc.M503515200 16048998

[48] Daleke MH, Ummels R, Bawono P, Heringa J, Vandenbroucke-Grauls CMJE, Luirink J, Bitter W. 2012. General secretion signal for the mycobacterial type VII secretion pathway. Proc Natl Acad Sci USA 109:11342–11347. doi:10.1073/pnas.1119453109 22733768 PMC3396530

[49] Champion PAD, Stanley SA, Champion MM, Brown EJ, Cox JS. 2006. C-terminal signal sequence promotes virulence factor secretion in *Mycobacterium tuberculosis*. Science 313:1632–1636. doi:10.1126/science.1131167 16973880

[50] Klein TA, Grebenc DW, Shah PY, McArthur OD, Dickson BH, Surette MG, Kim Y, Whitney JC. 2022. Dual targeting factors are required for LXG toxin export by the bacterial type VIIb secretion system. mBio 13:e02137–22. doi:10.1128/mbio.02137-22 36036513 PMC9600955

[51] Gkragkopoulou P, Garrett SR, Shah PY, Grebenc DW, Klein TA, Kim Y, Whitney JC. 2025. A widespread family of molecular chaperones promotes the intracellular stability of type VIIb secretion system–exported toxins. Proc Natl Acad Sci USA 122:e2503581122. doi:10.1073/pnas.2503581122 40953262 PMC12478183

[52] Ulhuq FR, Gomes MC, Duggan GM, Guo M, Mendonca C, Buchanan G, Chalmers JD, Cao Z, Kneuper H, Murdoch S, Thomson S, Strahl H, Trost M, Mostowy S, Palmer T. 2020. A membrane-depolarizing toxin substrate of the *Staphylococcus aureus* type VII secretion system mediates intraspecies competition. Proc Natl Acad Sci USA 117:20836–20847. doi:10.1073/pnas.2006110117 32769205 PMC7456083

[53] Zhang D, de Souza RF, Anantharaman V, Iyer LM, Aravind L. 2012. Polymorphic toxin systems: comprehensive characterization of trafficking modes, processing, mechanisms of action, immunity and ecology using comparative genomics. Biol Direct 7:18. doi:10.1186/1745-6150-7-18 22731697 PMC3482391

[54] Garrett SR, Mietrach N, Deme J, Bitzer A, Yang Y, Ulhuq FR, Kretschmer D, Heilbronner S, Smith TK, Lea SM, Palmer T. 2023. A type VII-secreted lipase toxin with reverse domain arrangement. Nat Commun 14:8438. doi:10.1038/s41467-023-44221-y 38114483 PMC10730906

[55] Colautti J, Kim Y, Whitney JC. 2025. Antibacterial ADP-ribosyl cyclase toxins inhibit bacterial growth by rapidly depleting NAD(P)+. J Biol Chem 301:110491. doi:10.1016/j.jbc.2025.110491 40680842 PMC12356396

[56] Teh WK, Ding Y, Gubellini F, Filloux A, Poyart C, Givskov M, Dramsi S. 2023. Characterization of TelE, a T7SS LXG effector exhibiting a conserved c-terminal glycine zipper motif required for toxicity. Microbiol Spectr 11:e0148123. doi:10.1128/spectrum.01481-23 37432124 PMC10434224

[57] Bai Q, Liu J, Zhao J, Pan X, Zhang Y, Wu Z, Ma J. 2025. Identifying a type of toxic effectors exported by the type VII secretion system to enhance competitive fitness in *Streptococcus suis*. Front Cell Infect Microbiol 15:1685307. doi:10.3389/fcimb.2025.1685307 41163853 PMC12558993

[58] Garrett SR, Mariano G, Dicks J, Palmer T. 2022. Homologous recombination between tandem paralogues drives evolution of a subset of type VII secretion system immunity genes in firmicute bacteria. Microb Genom 8:mgen000868. doi:10.1099/mgen.0.000868 35960642 PMC9484751

[59] Koskiniemi S, Garza-Sánchez F, Sandegren L, Webb JS, Braaten BA, Poole SJ, Andersson DI, Hayes CS, Low DA. 2014. Selection of orphan rhs toxin expression in evolved *Salmonella enterica* serovar Typhimurium. PLoS Genet 10:e1004255. 24675981 10.1371/journal.pgen.1004255 PMC3967940

[60] Bowman L, Palmer T. 2021. The type VII secretion system of *Staphylococcus*. Annu Rev Microbiol 75:471–494. https://www.annualreviews.org/content/journals/10.1146/annurev-micro-012721-123600 34343022 10.1146/annurev-micro-012721-123600 PMC7619418

[61] Shu X, Sun X, Wang K, Duan Y, Liu Y, Zhang R. 2025. LXG toxins of *Bacillus velezensis* mediate contact-dependent inhibition in a T7SS-dependent manner to enhance rhizosphere adaptability. IJMS 26:2592. doi:10.3390/ijms26062592 40141234 PMC11942605

[62] Liu Y, Shu X, Chen L, Zhang H, Feng H, Sun X, Xiong Q, Li G, Xun W, Xu Z, Zhang N, Pieterse CMJ, Shen Q, Zhang R. 2023. Plant commensal type VII secretion system causes iron leakage from roots to promote colonization. Nat Microbiol 8:1434–1449. doi:10.1038/s41564-023-01402-1 37248429

[63] Kobayashi K. 2021. Diverse LXG toxin and antitoxin systems specifically mediate intraspecies competition in *Bacillus subtilis* biofilms. PLoS Genet 17:e1009682. doi:10.1371/journal.pgen.1009682 34280190 PMC8321402

[64] Liang Z, Wu H, Bian C, Chen H, Shen Y, Gao X, Ma J, Yao H, Wang L, Wu Z. 2022. The antimicrobial systems of *Streptococcus suis* promote niche competition in pig tonsils. Virulence 13:781–793. doi:10.1080/21505594.2022.2069390 35481413 PMC9067509

[65] Lai L, Dai J, Tang H, Zhang S, Wu C, Qiu W, Lu C, Yao H, Fan H, Wu Z. 2017. *Streptococcus suis* serotype 9 strain GZ0565 contains a type VII secretion system putative substrate EsxA that contributes to bacterial virulence and a vanZ- like gene that confers resistance to teicoplanin and dalbavancin in *Streptococcus agalactiae*. Vet Microbiol 205:26–33. doi:10.1016/j.vetmic.2017.04.030 28622857

[66] Hsu C-C, Chuang Y-C, Hsu R-B, Chen J-W, Chia J-S, Shih Y-H, Kuo Y-M, Jung C-J. 2025. *Streptococcus gordonii* type VII secretion system substrate EsxA induces neutrophil extracellular trap formation in infective endocarditis. Sci Rep 15:43708. doi:10.1038/s41598-025-27543-3 41387757 PMC12701049

[67] Wu H, Wu Y, Ma J, Wu Z. 2026. A toxic effector of T7SS facilitates bacterial competition and virulence through membrane damage in *Streptococcus suis*. Vet Res 57:36. doi:10.1186/s13567-025-01706-6 41630062 PMC12955049

[68] Kamboyi HK, Paudel A, Shawa M, Sugawara M, Zorigt T, Chizimu JY, Kitao T, Furuta Y, Hang’ombe BM, Munyeme M, Higashi H. 2024. EsxA, a type VII secretion system-dependent effector, reveals a novel function in the sporulation of *Bacillus cereus* ATCC14579. BMC Microbiol 24:351. doi:10.1186/s12866-024-03492-1 39289639 PMC11406982

[69] Fyans JK, Bignell D, Loria R, Toth I, Palmer T. 2013. The ESX/type VII secretion system modulates development, but not virulence, of the plant pathogen *Streptomyces scabies*. Mol Plant Pathol 14:119–130. doi:10.1111/j.1364-3703.2012.00835.x 23009676 PMC6638804

[70] Sankey N, Merrick H, Singh P, Rogers J, Reddi A, Hartson SD, Mitra A. 2023. Role of the *Mycobacterium tuberculosis* ESX-4 secretion system in heme iron utilization and pore formation by PPE proteins. mSphere 8:e0057322. doi:10.1128/msphere.00573-22 36749044 PMC10117145

[71] Schulthess B, Bloes DA, Berger-Bächi B. 2012. Opposing roles of σB and σB-controlled SpoVG in the global regulation of esxA in *Staphylococcus aureus*. BMC Microbiol 12:17. doi:10.1186/1471-2180-12-17 22272815 PMC3313859

[72] Crosby HA, Tiwari N, Kwiecinski JM, Xu Z, Dykstra A, Jenul C, Fuentes EJ, Horswill AR. 2020. The *Staphylococcus aureus* ArlRS two-component system regulates virulence factor expression through MgrA. Mol Microbiol 113:103–122. doi:10.1111/mmi.14404 31618469 PMC7175635

[73] Lopez MS, Tan IS, Yan D, Kang J, McCreary M, Modrusan Z, Austin CD, Xu M, Brown EJ. 2017. Host-derived fatty acids activate type VII secretion in *Staphylococcus aureus*. Proc Natl Acad Sci USA 114:11223–11228. doi:10.1073/pnas.1700627114 28973946 PMC5651732

[74] Casabona MG, Kneuper H, Alferes de Lima D, Harkins CP, Zoltner M, Hjerde E, Holden MTG, Palmer T. 2017. Haem-iron plays a key role in the regulation of the Ess/type VII secretion system of *Staphylococcus aureus* RN6390. Microbiology (Reading) 163:1839–1850. doi:10.1099/mic.0.000579 29171824 PMC5845736

[75] Finn JP, Luzinski C, Burton BM. 2024. Differential expression of the yfj operon in a *Bacillus subtilis* biofilm. Appl Environ Microbiol 90:e01362–24. doi:10.1128/aem.01362-24 39436054 PMC11577775

[76] Spencer BL, Deng L, Patras KA, Burcham ZM, Sanches GF, Nagao PE, Doran KS. 2019. Cas9 contributes to group B streptococcal colonization and disease. Front Microbiol 10:1930. doi:10.3389/fmicb.2019.01930 31497003 PMC6712506

[77] Avican K, Aldahdooh J, Togninalli M, Mahmud A, Tang J, Borgwardt KM, Rhen M, Fällman M. 2021. RNA atlas of human bacterial pathogens uncovers stress dynamics linked to infection. Nat Commun 12:3282. doi:10.1038/s41467-021-23588-w 34078900 PMC8172932

[78] Prasopthum P, Thanongsaksrikul J, Khantisitthiporn O, Tongtawe P, Arechanajan B, Srimanote P. 2025. Serotype 2 *Streptococcus suis* growth inhibition mediated by intraspecies contact-dependent mechanism. Sci Rep 15:19565. doi:10.1038/s41598-025-04541-z 40467950 PMC12137939

[79] Burcham LR, Bath JR, Werlang CA, Lyon LM, Liu N, Evans C, Ribbeck K, Doran KS. 2022. Role of MUC5B during group B streptococcal vaginal colonization. mBio 13:e00039–22. doi:10.1128/mbio.00039-22 35323039 PMC9040740

[80] Sitkiewicz I, Green NM, Guo N, Bongiovanni AM, Witkin SS, Musser JM. 2009. Transcriptome adaptation of group B *Streptococcus* to growth in human amniotic fluid. PLoS One 4:e6114. doi:10.1371/journal.pone.0006114 19568429 PMC2700258

[81] Burcham LR, Akbari MS, Alhajjar N, Keogh RA, Radin JN, Kehl-Fie TE, Belew AT, El-Sayed NM, McIver KS, Doran KS. 2022. Genomic analyses identify manganese homeostasis as a driver of group B streptococcal vaginal colonization. mBio 13:e0098522. doi:10.1128/mbio.00985-22 35658538 PMC9239048

[82] Cook LCC, Hu H, Maienschein-Cline M, Federle MJ. 2018. A vaginal tract signal detected by the group B *Streptococcus* SaeRS system elicits transcriptomic changes and enhances murine colonization. Infect Immun 86:e00762-17. doi:10.1128/IAI.00762-17 29378799 PMC5865029

[83] Sullivan MJ, Prince D, Goh KGK, Katupitiya L, Gosling D, Crowley MR, Crossman DK, Ulett GC. 2023. Dual RNA sequencing of group B *Streptococcus*-infected human monocytes reveals new insights into host-pathogen interactions and bacterial evasion of phagocytosis. Sci Rep 13:2137. doi:10.1038/s41598-023-28117-x 36747074 PMC9902490

[84] Sullivan MJ, Goh KGK, Ulett GC. 2022. Regulatory cross-talk supports resistance to Zn intoxication in *Streptococcus*. PLoS Pathog 18:e1010607. doi:10.1371/journal.ppat.1010607 35862444 PMC9345489

[85] Tiffany CR, Bäumler AJ. 2019. Dysbiosis: from fiction to function. Am J Physiol Gastrointest Liver Physiol 317:G602–G608. doi:10.1152/ajpgi.00230.2019 31509433 PMC6879887

[86] Winter SE, Winter MG, Xavier MN, Thiennimitr P, Poon V, Keestra AM, Laughlin RC, Gomez G, Wu J, Lawhon SD, Popova IE, Parikh SJ, Adams LG, Tsolis RM, Stewart VJ, Bäumler AJ. 2013. Host-derived nitrate boosts growth of *E. coli* in the inflamed gut. Science 339:708–711. doi:10.1126/science.1232467 23393266 PMC4004111

[87] Winter Sebastian E., Thiennimitr P, Winter MG, Butler BP, Huseby DL, Crawford RW, Russell JM, Bevins CL, Adams LG, Tsolis RM, Roth JR, Bäumler AJ. 2010. Gut inflammation provides a respiratory electron acceptor for *Salmonella*. Nature 467:426–429. doi:10.1038/nature09415 20864996 PMC2946174

[88] Kaambo E, Africa C, Chambuso R, Passmore J-AS. 2018. Vaginal microbiomes associated with aerobic vaginitis and bacterial vaginosis. Front Public Health 6:78. doi:10.3389/fpubh.2018.00078 29632854 PMC5879096

[89] Dubé-Zinatelli E, Cappelletti L, Ismail N. 2025. The vaginal microbiome in bacterial vaginosis: pathogenesis, reproductive impacts, and emerging therapies. J Reprod Immunol 172:104804. doi:10.1016/j.jri.2025.104804 41275619

[90] Aly KA, Anderson M, Ohr RJ, Missiakas D. 2017. Isolation of a membrane protein complex for type VII secretion in *Staphylococcus aureus*. J Bacteriol 199:e00482-17. doi:10.1128/JB.00482-17 28874412 PMC5686593

[91] Collars OA, Hernandez RL, Weaver SD, Prest RJ, Manu C, Viswanathan G, Cronin RM, Jones BS, Tobin DM, Champion MM, Champion PA. 2026. pH-responsive substrate switching in mycobacterial type VII ESX secretion. mSphere 11:e0005626. doi:10.1128/msphere.00056-26 42053305 PMC13203967

[92] Bobrovskyy M, Willing SE, Schneewind O, Missiakas D. 2018. EssH peptidoglycan hydrolase enables *Staphylococcus aureus* type VII secretion across the bacterial cell wall envelope. J Bacteriol 200:e00268-18. doi:10.1128/JB.00268-18 30082459 PMC6153663

[93] Agyen R, Powell I, McNair M, Missiakas D, Bobrovskyy M. 2026. Type VIIb secretion system recruits the dedicated cell wall hydrolase EssH to enable effector secretion by *Staphylococcus aureus*. mBio 17:e0319625. doi:10.1128/mbio.03196-25 41744680 PMC13059805

[94] Klein TA, Grebenc DW, Gandhi SY, Shah VS, Kim Y, Whitney JC. 2021. Structure of the extracellular region of the bacterial type VIIb secretion system subunit EsaA. Structure 29:177–185. doi:10.1016/j.str.2020.11.002 33238147

[95] Sørensen AL, Nagai S, Houen G, Andersen P, Andersen AB. 1995. Purification and characterization of a low-molecular-mass T-cell antigen secreted by *Mycobacterium tuberculosis*. Infect Immun 63:1710–1717. doi:10.1128/iai.63.5.1710-1717.1995 7729876 PMC173214

[96] Alhajjar N, Chatterjee A, Spencer BL, Burcham LR, Willett JLE, Dunny GM, Duerkop BA, Doran KS. 2020. Genome-wide mutagenesis identifies factors Involved in *Enterococcus faecalis* vaginal adherence and persistence. Infect Immun 88:e00270–20. doi:10.1128/IAI.00270-20 32778611 PMC7504943

[97] Taylor JC, Gao X, Xu J, Holder M, Petrosino J, Kumar R, Liu W, Höök M, Mackenzie C, Hillhouse A, Brashear W, Nunez MP, Xu Y. 2021. A type VII secretion system of *Streptococcus gallolyticus* subsp. gallolyticus contributes to gut colonization and the development of colon tumors. PLoS Pathog 17:e1009182. doi:10.1371/journal.ppat.1009182 33406160 PMC7815207

[98] Spencer BL, Nguyen DT, Marroquin SM, Gapin L, O’Brien RL, Doran KS. 2025. Characterization of the cellular immune response to group B streptococcal vaginal colonization. J Innate Immun 17:1–22. doi:10.1159/000548044 PMC12659609 41047837

[99] Swietnicki W, Carmany D, Retford M, Guelta M, Dorsey R, Bozue J, Lee MS, Olson MA. 2011. Identification of small-molecule inhibitors of *Yersinia pestis* type III secretion system YscN ATPase. PLoS One 6:e19716. doi:10.1371/journal.pone.0019716 21611119 PMC3097197

[100] Arya T, Oudouhou F, Casu B, Bessette B, Sygusch J, Baron C. 2019. Fragment-based screening identifies inhibitors of ATPase activity and of hexamer formation of Cagα from the *Helicobacter pylori* type IV secretion system. Sci Rep 9:6474. doi:10.1038/s41598-019-42876-6 31019200 PMC6482174

[101] Bzdzion L, Krezel H, Wrzeszcz K, Grzegorek I, Nowinska K, Chodaczek G, Swietnicki W. 2017. Design of small molecule inhibitors of type III secretion system ATPase EscN from enteropathogenic *Escherichia coli*. Acta Biochim Pol 64:49–63. doi:10.18388/abp.2016_1265 27864920

[102] Zhou H, Du H, Zhang H, Shen H, Yan R, He Y, Wang M, Zhu X. 2013. EsxA might as a virulence factor induce antibodies in patients with *Staphylococcus aureus* infection. Braz J Microbiol 44:267–271. doi:10.1590/S1517-83822013005000019 24159314 PMC3804208

[103] Deng J, Wang X, Zhang B-Z, Gao P, Lin Q, Kao R-T, Gustafsson K, Yuen K-Y, Huang J-D. 2019. Broad and effective protection against *Staphylococcus aureus* is elicited by a multivalent vaccine formulated with novel antigens. mSphere 4:e00362–19. doi:10.1128/mSphere.00362-19 31484738 PMC6731528

[104] Zhang BZ, Hua YH, Yu B, Lau CCY, Cai JP, Zheng SY, Yam WC, Kao RYT, Sze KH, Zheng BJ, Yuen KY, Huang JD. 2015. Recombinant ESAT-6-like proteins provoke protective immune responses against invasive *Staphylococcus aureus* disease in a murine model. Infect Immun 83:339–345. doi:10.1128/IAI.02498-14 25368117 PMC4288882

[105] Sugrue I, Ross RP, Hill C. 2024. Bacteriocin diversity, function, discovery and application as antimicrobials. Nat Rev Microbiol 22:556–571. doi:10.1038/s41579-024-01045-x 38730101 PMC7616364

